# Stopping female feticide in India: the failure and unintended consequence of ultrasound restriction

**DOI:** 10.7189/jogh.07.010304

**Published:** 2017-06

**Authors:** Sheida Tabaie

**Affiliations:** Weill Cornell Medical College, Department of Anesthesiology, New York, New York, USA

Female feticide is an alarmingly common practice in India, as evidenced by the most recent Indian national census in 2011 indicating only 914 females for every 1000 males in the zero to six years age range [1]. The root cause of female feticide, a form of structural violence against women, is multifaceted and complex. As the Indian Ministry of Health and Family Welfare states in its 2006 annual report, “the social, cultural and religious fibre of India is predominantly patriarchal, comprehensively contributing to the secondary status of women” [2]. The high rates of female feticide reflect this secondary status. A lower earning capacity, the patrilineal social structure dictating inheritance, and the widespread practice of dowry contribute to the diminished position of women [2]. Improvement in the socioeconomic conditions in India has done little to raise the status of women. Recent evidence reveals that sex selection remains common among the affluent and educated in India [3].

In an attempt to curb female feticide, the Indian Government enacted the Pre–Natal Diagnostic Techniques (PNDT) Act of 1994, which prohibits sex selection and regulates prenatal diagnostic techniques to prevent their misuse. To this end, the government established a bureaucracy controlling the sale and regulating the use of ultrasound machines, a key diagnostic modality used to facilitate sex–selective abortions. Following the initial implementation of the PNDT Act, a further decline in the 2001 Indian national census sex ratio prompted the passage of an amendment, the Pre–Conception and Pre–Natal Diagnostic Techniques (PCPNDT) Act of 2003, which addressed pre–conception sex determination and strengthened enforcement of the PNDT Act.

The continual decline in the sex ratio with each national census since the inception of the PNDT Act calls into question its effectiveness. Census data shows that the sex ratio declined from 945 females for every 1000 males in the zero to six years of age range in 1991 to 927 females for every 1000 males in 2001 to 914 females for every 1000 males in 2011 [1,2]. A well–intentioned legal tool based on the principle of deterrence, the PCPNDT Act also suffers from weak implementation [4]. In its 2010 review of the PCPNDT Act, the Public Health Foundation of India acknowledges, “Data till 2006 reveal that as many as 22 of the 35 states in India had not reported a single violation of the Act since it came into force” [4].

It is difficult to ascertain whether the failure of the Act to substantially reduce female feticide is due to ineffectiveness or improper implementation. However, the unintended consequence of the PCPNDT Act is clear. To prevent misuse, the Act created a system wherein all individuals and institutions must register under the Act to legally purchase an ultrasound, regardless of whether the intended use involves prenatal diagnostics. Furthermore, all ultrasound practitioners, whether those using ultrasound for echocardiography or those using ultrasound for placement of central vascular access, are mandated to register with the Act. The system has resulted in onerous, time–consuming registration paperwork that discourages medical professionals outside of radiology from using ultrasound. By creating these bureaucratic barriers to ultrasound use unrelated to prenatal diagnostics, the Act restricts the medical practice of a broad range of physicians.

**Figure:**
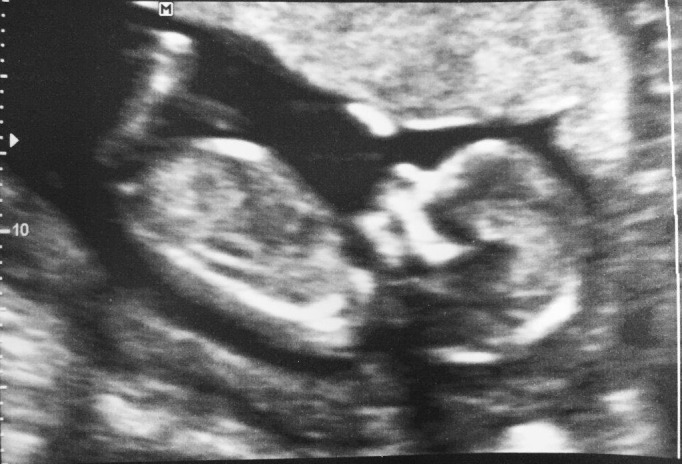
Photo: Photo from the personal collection of Gunisha Kaur, used with permission.

Affecting specialties as diverse as trauma, cardiology, and anesthesiology, advances in ultrasound have transformed the practice of medicine in the years since the PNDT Act was first passed. For example, in 1994, the year the PNDT Act was enacted, anesthesiologists rarely used ultrasound. Today, it is a staple of daily anesthesiology practice as a procedural and diagnostic tool, and it is the standard of care for peripheral nerve blocks and central vascular access. Beyond the operating theatres, ultrasound is commonplace in both operating theaters and intensive care units in the form of lung ultrasonography, transthoracic echocardiography, and transesophageal echocardiography.

To determine awareness of the PCPNDT Act and to gauge the accessibility of ultrasound, an anonymous survey was performed in the anesthesiology departments of hospitals in the State of Punjab, which has one of the highest rates of female feticide in the country [1]. The survey, sponsored by the Department of Anesthesiology at Weill Cornell Medical College as part of its Global Health Initiative, was conducted in person during February and March of 2016 at eight Punjabi tertiary care hospitals associated with medical colleges, both private and government–run. Anonymity was necessary to protect participants from potential legal ramifications of ultrasound use that was not compliant with PCPNDT regulations. At each hospital, a five–question survey was administered to a senior member of the anesthesiology department. The five questions addressed the following: awareness of the PCPNDT Act, number of anesthesiologists in the department with PCPNDT Act certification to use an ultrasound, number of PCPNDT registered ultrasounds available for use in the operating theaters, number of PCPNDT registered ultrasounds available for use in the intensive care unit, and incorporation of ultrasound in the medical school curriculum.

All eight anesthesiologists were aware of the existence and the purpose of the PCPNDT Act. Yet, none were able to articulate the Act’s specific regulations pertaining to their practice. Only two of the eight anesthesiology departments had PCPNDT certification for anesthesiologists in the department to use an ultrasound. These two departments had ultrasound available in both the operating theaters and the intensive care units. However, one department without PCPNDT certification for its anesthesiologists had ultrasound available in the operating theaters. The department without proper registration did not appear to fully grasp the potential legal consequences of this breach of the PCPNDT Act. None of the anesthesiologists were involved in teaching ultrasound to medical students.

It is striking that the vast majority of anesthesiologists in these tertiary care centers did not have access to an ultrasound and that one third of those that did have access to ultrasound were non–compliant with PCPNDT regulations. All of the anesthesiologists surveyed expressed frustration at this inaccessibility, echoing the sentiment that care could be improved if this important tool was more widely available. One anesthesiologist cited a delay of nearly two years to obtain an ultrasound due to the PCPNDT registration process. How can the Indian anesthesiology community, or other medical disciplines that rely heavily on ultrasound, uphold the standard of care when access to this critical imaging modality is limited? Given that the census data has not shown a significant decrease in female feticide since the inception of the Act in 1994, it is difficult to justify the sacrifice in patient care.

The PCPNDT Act oversimplifies a complex problem by placing the moral and legal onus on physicians instead of on patients committing female feticide and the families supporting them. As the former Indian Health Minister Harsh Vardhan aptly stated in October 2014: “It is clear that the focus on the providers of sex selection services has not worked through 20 years. We need to go into the root cause and build up a social movement” [5]. The “Save the girl child, educate the girl child” campaign and free government education for girls in Punjab are steps in the right direction. However, given the concentration of sex imbalance among the highly educated, it follows that a nuanced, multidimensional approach extending beyond educational opportunities is required to achieve social parity for women.

Indian society as a whole shares the collective responsibility for female feticide. Yet, Indian physicians are in a unique and powerful position. They have the capability to disrupt the structural violence against women by refusing to participate in female feticide, a clear breach of medical ethics, as defined by the principles of beneficence and nonmaleficence. Despite its shortcomings, the PCPNDT Act is a well-intentioned piece of social legislation that strengthens the practice of medical ethics by providing a legal incentive for Indian physicians to uphold their obligations [4]. While the PCPNDT Act succeeds in acknowledging and drawing attention to a grave societal problem, its failure to significantly curb female feticide and its unintended consequence cannot be overlooked. The burdensome restrictions on ultrasound, which prevent Indian physicians from accessing a valuable imaging modality, have not translated into the social change intended by the PCPNDT Act. Ultimately, ending female feticide will require a solution as multifaceted and complex as the underlying root causes.
